# Psychiatric comorbidity in chronic urticaria patients: a systematic review and meta-analysis

**DOI:** 10.1186/s13601-019-0278-3

**Published:** 2019-08-23

**Authors:** Gerasimos N. Konstantinou, George N. Konstantinou

**Affiliations:** 1 Department of Psychiatry, 251 Hellenic Airforce V. A. General Hospital, Athens, Greece; 2 Department of Allergy and Clinical Immunology, 424 General Military Training Hospital, 11 Eleftheriou Benizelou Street, Kalamaria, 55 133 Thessaloniki, Greece

**Keywords:** Psychiatric disorders, Psychopathology, Prevalence, Anxiety, Depression, Stress

## Abstract

**Background:**

Dermatological illness can affect the quality of life and may coexist with psychiatric disorders.

**Objective:**

The aim of this review was to systematically evaluate the published evidence of any psychiatric disorders that may coexist with chronic urticaria (CU) and any effect psychiatric interventions may have on CU.

**Methods:**

Following the Cochrane guidance, we conducted a systematic literature search using web-based search engines provided by PubMed (for Medline database), Google Scholar and Scopus for studies that have investigated the existence of psychiatric comorbidity in patients with CU. To be included, a study had to possess features, such as: (1) distinction between chronic urticaria and allergic conditions, (2) direct collection of diagnostic psychiatric data by using clinical interview and standardized questionnaires, (3) International Classification of Disorders criteria or the Diagnostic and Statistical Manual of Mental Disorders criteria for the diagnosis of mental disorders, and (4) manuscripts written or published in the English language. Unpublished research and research in progress were not included. All the eligible studies were scrutinized for any reported psychiatric interventions that had any effect on CU. The systematic review has been registered on PROSPERO (registration number CRD42019122811) and was conducted following the Preferred Reporting Items for Systematic Reviews and Meta-Analysis (PRISMA).

**Results:**

Twenty-five studies were identified. Almost one out of three CU patients have at least one underlying psychiatric disorder. None of the studies clarified whether the psychiatric disorders pre-existed the CU onset, and no association was found between CU severity and duration, and psychological functioning. Only one case report and two case series mentioned that treatment of psychiatric disorders with either anti-depressants, anti-anxiety drugs or psychological interventions might result in improvement of urticaria.

**Conclusions:**

Patients with CU frequently experience psychiatric disorders. This highlights the need for a multidisciplinary therapeutic approach involving prompt recognition and management of any potential psychiatric disorder in addition to urticaria treatment. Further studies are needed to assess whether psychiatric disorders coexist with CU independently or follow urticaria onset and whether any psychological or psychiatric intervention may help in CU control.

## Introduction

Chronic urticaria (CU) is a troublesome entity that presents with wheals, angioedema or both almost daily for at least 6 consecutive weeks. Both wheals and angioedema are characterized by superficial dermal swelling favoring the papillary dermis in the case of the wheal formation, and the deep dermis and subcutis in the case of the angioedema. The urticarial lesions may be intensively pruritic, and the angioedema may present with mild pain and burning sensation [1], symptoms that affect the patient’s quality of life significantly and may account for stress, sleep disorders, negative self-image, disability in social functions and adverse emotions such as anger and low mood/sadness [2–4]. The prevalence of CU in the general population has been estimated to range from 0.5 to 5% and has consequently been considered as a non-negligible clinical entity [5].

The psychological effects that several dermatological entities and allergic conditions may cause have long been speculated or even recognized [6–9]. Back in 1940, Clarke [10] lectured on the interhospital conference held at the Utica State Hospital at Utica, NY (*sic*) “*The mental effects of allergy have received little attention, although nervous symptoms are so common in association with the allergic diseases that until recently asthma, urticaria, angioneurotic edema, and migraine were thought to be primarily diseases of the nervous system*”. More recently published evidence suggests a clear association between CU and psychiatric disorders, most commonly depression and anxiety [11–13].

Most of the physicians that consult CU patients are not aware that urticaria and several psychological or psychiatric disorders may be interconnected, except urticaria experts that approach these patients in the context of a bio-psycho-social model [14, 15]. In the absence of any existing systematic review of the literature approaching this fact, we performed a systematic review of the literature with the intention to examine the prevalence of any psychiatric disorder among patients with CU, any potential association that may exist and the role any psychiatric intervention may have in urticaria control and treatment. Furthermore, we investigated the literature for evidence suggestive of whether psychiatric disorders pre-exist, co-exist or follow CU and if there is any association between CU severity and duration or severity of the psychological functioning.

## Method

This systematic literature review was conducted and reported following the Preferred Reporting Items for Systematic Reviews and Meta-Analysis (PRISMA) [16]. The Cochrane guidance for non-randomized studies was followed for meta-analysis of the results. The protocol of this systemic review has been registered on PROSPERO with registration number CRD42019122811.

An a priori set of inclusion criteria was predefined. To be included, a study had to possess features, such as: (1) clear distinction between urticaria and other allergies, (2) clear distinction between acute and chronic urticaria, (3) utilization of appropriate clinical interviews, standardized questionnaires and criteria for psychopathology diagnosis and standard diagnostic nomenclature for mental and behavioural disorders [including the International Statistical Classification of Diseases and Related Health Problems (ICD) and the Diagnostic and Statistical Manual of Mental Disorders (DSM)], and (5) manuscripts written or published in English language. Unpublished research and research in progress were not included. The references of eligible publications were scrutinized to identify additional possible studies.

### Criteria for considering studies for this review

#### Types of studies

Included studies were randomized clinical trials (RCT), controlled clinical trials, cohort studies, case–control studies, case series and case reports relevant to CU patients (adults) that investigated the comorbidity of psychiatric disorders and symptoms.

#### Types of participants

The primary outcome of interest was the assessment of the prevalence of psychiatric comorbidities in CU patients by any validated measure. Secondary outcomes were measures of disease severity and psychiatric interventions on CU patients. The outcome terminologies presented in this report are those used in the original publications.

### Search strategy for identification of studies

A comprehensive search strategy, including all the relevant synonyms for the central concepts, was developed. Web-based search engines provided by PubMed (for Medline database), Google Scholar and Scopus were utilized. Key phrases used for the research were: “psychiatric disorders and chronic urticaria”, “psychiatric comorbidity and allergy”, “psychiatry and urticaria”, “depression and urticaria”, “anxiety and urticaria”, “psychiatric morbidity in dermatology”, “urticaria and comorbidity”, “psychiatry and dermatology”, “psychiatry and allergology”, “psychodermatology”, and “stress and urticaria”. The last publication month that has been systematically reviewed was December 2018.

### Data collection

Two independent reviewers assessed all titles and abstracts, extracted all data, and assessed quality. A highly sensitive search strategy identified all published articles. The full text of all potentially eligible studies was assessed for eligibility against the predefined inclusion criteria. The reference list from these articles, including all relevant review articles, was analyzed for other potentially relevant studies not identified in the data-based search. All the eligible studies were scrutinized for any reported psychiatric interventions that had any effect on CU. All of the included studies were discussed and approved by the review team.

### Assessment of risk of bias in included studies

The risk of bias tool described in the Cochrane Handbook for Systematic Reviews for Interventions was used to appraise the studies.

## Results

**Fig. 1:**
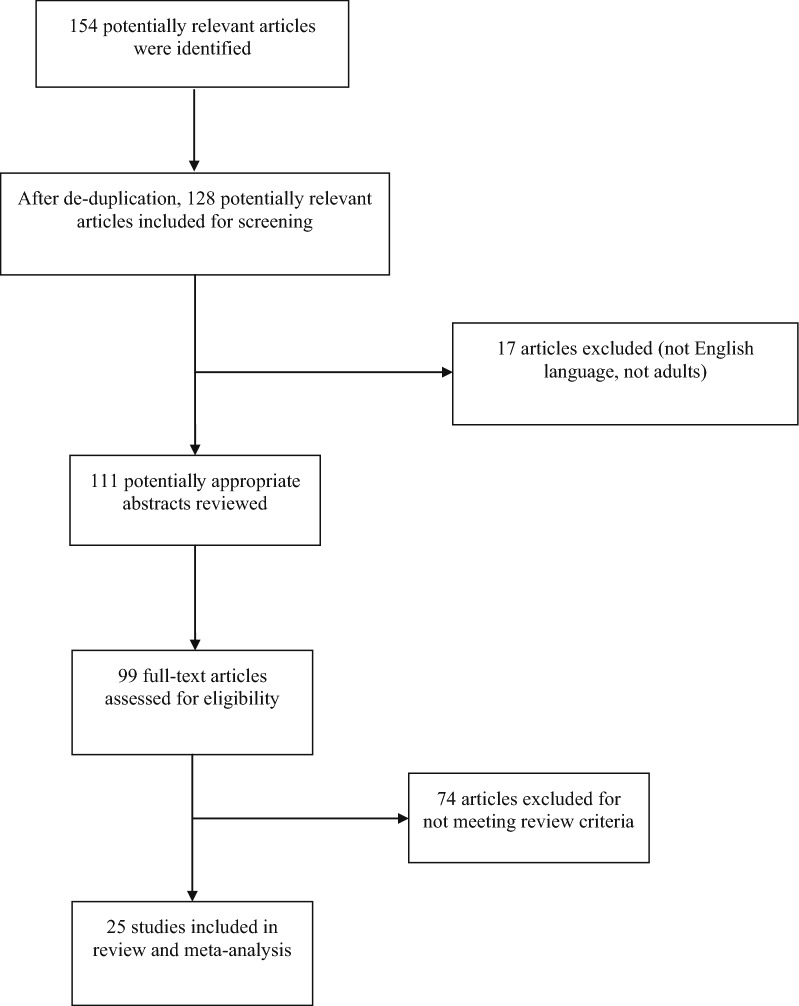
Preferred reporting items for systematic reviews and meta-analysis (PRISMA) flow diagram

Our search identified 154 potentially relevant published papers; 25 of them (11 cohort studies, 11 case–control studies, 2 case series, and 1 case report) satisfied our inclusion criteria and were included in our meta-analysis (Fig. 1). Some of these studies did use a control group (not necessarily comprised with healthy individuals) to compare psychopathologic comorbidities among CU patients, and some of them did not examine psychopathology as a whole but focused on specific psychiatric entities (e.g., studies that focused only on depression and did not examine the coexistence of any other psychiatric disorder) (Table 1).

**Table 1:** Psychiatric comorbidity among chronic urticaria patients: all studies

Study	Type of study	Patients	Control group
I. Psychiatric comorbidity in CU patients (studies without a control group)			
1. Juhlin [18]	Cohort study	330 CU patients	No
2. Pulimood et al. [19]	Cohort study	20 CU patients	No
3. Picardi et al. [20]	Cohort study	29 CU patients	No
4. Picardi et al. [21]	Cohort study	16 CU patients	No
5. Staubach et al. [22]	Cohort study	100 CU patients	No
6. Mehta et al. [23]	Cohort study	50 CU patients	No
7. Sorour et al. [24]	Cohort study	110 CU patients	No
II. Psychiatric comorbidity in CU patients (studies with healthy individuals as a control group)			
8. Uguz et al. [26]	Case–control	89 CU patients	60 individuals
9. Atefi et al. [25]	Case–control	30 CU patients	30 individuals
10. Chu et al. [17]	Case–control	177,879 CU patients	996,356 individuals
11. Staubach et al. [27]	Case–control	100 CU patients	96 individuals
12. Ozkan et al. [28]	Case–control	84 CU patients	75 individuals
13. Lapi et al. [29]	Case–control	3489 CU patients	1,714,746 individuals
III. Psychiatric comorbidity in CU patients (studies with control subjects other than healthy individuals)			
14. Calıkusu et al. [31]	Case–control	31 CU patients	31 patients with psychogenic excoriation (PE)
15. Yang et al. [30]	Case–control	75 CU outpatients	133 patients with tinea pedis
IV. Comorbidity with specific psychiatric disorders in CU patients			
16. Demet et al. [33]	Cohort study	7 CU	No
17. Sukan et al. [34]	Case–control	50 CU patients, 50 vitiligo patients	50 individuals
18. Chung et al. [32]	Case–control	100 CU patients	60 patients with allergy
19. Bashir et al. [36]	Cohort study	3 CU patients	No
20. Tuna et al. [35]	Case–control	130 CU patients	100 individuals
V. Case reports–case series—psychiatric interventions in CU patients			
21. Hashiro [37]	Case report	1 CU patient with anxiety-depressive disorder	No
22. Gupta et al. [39]	Case series	5 patients with PTSD diagnosed with CU	No
23. Gupta et al. [38]	Case series	2 CU patients with panic disorder	No
VI. Other studies			
24. Seyhan et al. [40]	Cohort study	636 dermatologic patients	No
25. Perugi et al. [41]	Cohort study	347 patients with bipolar disorder	No

The overall prevalence of any psychiatric comorbidity among CU patients independently of whether studies had or didn’t have a control group was estimated to be 31.61%, (data from Tables 2 and 3). The biggest study among them by Chu et al. [17] from Taiwan was based on an international database for health insurance. Because the sample in this study was large and the particularities of the examined individuals were expected to increase heterogeneity substantially, the pooled prevalence was calculated, as well, without this study and was found to be exactly the same (31.61%).

**Table 2:** Psychiatric comorbidity in chronic urticaria patients—studies not including a control group

		CU patients	CU patients with psychiatric comorbidity
Juhlin [18]			
Cohort study		330	53
Pulimood et al. [19]			
Cohort study		20	15
Picardi et al. [20]			
Cohort study		29	10
Staubach et al. [22]			
Cohort study		100	48
Total		479	126
*Pooled prevalence*			*26.3%*

**Table 3:** Psychiatric comorbidity in chronic urticaria (CU) patients—studies including a control group

		Chronic urticaria patients		Control individuals	
		All	With psychiatric comorbidity	All	With psychiatric comorbidity
Uguz et al. [26]					
Case–control study		89	44	60	8
Atefi et al. [25]					
Case–control study		30	19	30	14
Chu et al. [17]					
Case–control study		177,879	56,195	996,356	45,449
Total		177,998	56,258	996,446	45,471
		119^#^	63^#^	90^#^	22^#^
*Pooled prevalence*			31.6%*		4.6%*
			*52.9%*^#,^ **		*24.4%*^#,^ **^`^

* Pearson’s χ^2^: p-value < 0.001

** Pearson’s χ^2^: p-value = 0.006

^#^After excluding Chu et al. study

The pooled prevalence of each psychiatric disorder among CU patients, categorized according to Diagnostic and Statistical Manual of Mental Disorders (DSM-5), are presented in Table 4 (data listed for disorders referred in at least two different published articles). The most prevalent were found to be sleep–wake disorders (36.7%), followed by anxiety disorders (30.6%), mood disorders (29.4%), trauma and stressor-related disorders (17.3%), somatic symptom and related disorders (17.2%), obsessive–compulsive and related disorders (9.3%) and substance-related and addictive disorders (4%).

**Table 4:** Pooled prevalence of psychiatric disorders among chronic urticaria (CU) patients, categorized according to Diagnostic and Statistical Manual of Mental Disorders (DSM-5) (data listed for disorders referred in at least two different published articles)

DSM-5 classification	Patients N/all CU patients		Control^a^ N/all healthy controls		p-value*
	Prevalence per study	Pooled prevalence (%)	Prevalence per study	Pooled prevalence (%)	
Sleep–wake disorders (e.g., insomnia disorder, restless legs syndrome)	57/110 [24] 31/130 [35]	88/240 (*36.7%*)	12/100 [35]	(*12%*) based only of ref. [35]	*<* *0.001*
Anxiety disorders (e.g. generalized anxiety disorder, phobias)	30/100 [22] 2/50 [23] 48/110 [24] 24/30 [25] 39/89 [26] 25/100 [27] 10/84 [28] 4/31 [31] 15/50 [34]	197/644 (*30.6%*)	13/30 [25] 7/64 [26]	20/94 (*21.3%*)	0.16^b^
Mood disorders (major depressive disorder, dysthymic disorder)	12/20 [19] 17/100 [22] 16/50 [23] 39/110 [24] 15/30 [25] 18/89 [26] 11/100 [27] 36/84 [28] 6/31 [31] 23/50 [34] 2/3 [36]	195/664 (*29.4%*)	5/30 [25] 3/64 [26]	8/94 (*8.5%*)	*0.001*
Trauma and stressor-related disorders (e.g. posttraumatic stress disorder, adjustment disorder)	5/100 [22] 1/31 [31] 34/100 [32]	40/231 (*17.3%*)	–	–	–
Somatic symptom and related disorders (e.g., somatic symptom disorder, conversion disorder)	17/100 [22] 21/30 [25] 11/100 [27] 5/84 [28]	54/314 (*17.2%*)	12/30 [25]	(*40%*) based only of ref. [25]	*0.020* ^c^
Obsessive–compulsive and related disorders	4/100 [22] 1/50 [23] 3/110 [24] 23/89 [26] 3/31 [31] 2/7 [33]	36/387 (*9.3%*)	1/64 [26]	(*1.6%*) based only of ref. [26]	*0.045*
Substance-related and addictive disorders (e.g., alcohol use disorder)	5/100 [22] 1/50 [23]	6/150 (*4%*)	–	–	–

*N* number of patients with psychiatric disorders

*Comparisons based on Pearson’s χ^2^

^a^Only studies comparing the prevalence of psychiatric disorders in CU patients and healthy controls are listed

^b^Non-statistically significant result attributed to the high prevalence of psychiatric entities among control group in reference 25 (46.6%)

^c^Statistically significant result attributed to the high prevalence of psychiatric entities among control group in reference 25 (46.6%)

### I. Psychiatric comorbidities in patients with chronic urticaria (studies without a control group)

#### Description of studies

Seven studies examined psychiatric comorbidity in patients with CU without providing a control group [18–24].

Four of them clearly mentioned both the total number of included CU patients and how many of these patients had at least one psychiatric comorbidity (sum 479 and 126, respectively, pooled prevalence 26.3%, Table 2). From the remaining three studies, two mentioned the absolute number of patients suffering from specific psychiatric disorders, without clarifying whether any of these patients had more than one of them [23, 24]. Since psychiatric illnesses may very often co-exist, in these two studies, it was not possible to calculate the exact psychiatric comorbidity among the examined CU patients.

The last study by Picardi et al. [21] referred to the prevalence and correlates of suicidal ideation among patients with skin diseases. Although suicidal ideation is not categorized as a specific psychiatric disorder, according to DSM, it is closely related to the majority of mental illness.

#### Main findings

In 1981, Juhlin [18], after studying 330 patients with CU reported that 16% (n = 53) of them had a psychiatric history of taking relevant medications. No individual psychiatric diagnoses were reported.

Pulimood et al. [19] determined the prevalence and nature of psychiatric morbidity among 1073 dermatological inpatients. Among them, 20 patients were suffering from CU, 15 of whom were comorbid with psychiatric pathology.

In 2000 Picardi et al. [20] studied the prevalence of psychiatric disorders in 2579 dermatological outpatients. Among the 29 patients with CU, the percentage of psychiatric cases was 34.5% (n = 10).

Although suicidal ideation is not yet an official psychiatric disorder according to DSM, Picardi et al. [21] sought to estimate the prevalence of suicidal ideation among 466 patients with dermatologic conditions and found that was present among 16 patients with urticaria (prevalence 18.8%).

Staubach et al. [22] in 2011 assessed the prevalence and spectrum of mental disorders and determined levels of emotional distress in patients with CU. A total sample of 100 patients with CU was screened for enrollment in this study and was investigated for mental disorders. Forty-eight of them were found to have one or more mental disorders. The most common mental disorders identified were anxiety (30%), followed by depressive and somatoform disorders (17% each). Agoraphobia was found to be the most frequent anxiety disorder in patients with CU (15%).

In 2007, Mehta et al. [23] evaluated the psychiatric illness in psoriasis vulgaris and CU patients. In 50 CU patients, most common psychiatric co-morbidity was depression (30%) followed by suicidality (12%), panic disorder (4%), obsessive–compulsive disorder (2%), alcohol abuse and dependence psychotic disorder and mood disorder with psychotic features (2%). Unfortunately, the total number of patients suffering from any psychiatric disorder was not mentioned.

Similarly, in 2017, Sorour et al. [24] performed an analysis of psychiatric disorders associated with chronic dermatologic diseases among a group of Egyptian patients. This study included 110 patients with CU and anxiety was found in 43.64% (n = 48), depression in 35.45% (n = 39), suicide ideation in 21 (19.09%), sleep disorders in 57 (51.82%), obsessive–compulsive diseases in 3 (2.73%) and finally sexual disorders in 24 of them (21.82%).

### II. Psychiatric comorbidities in patients with chronic urticaria (studies with healthy individuals as a control group)

#### Description of studies

Three studies examined the comorbidity of CU with psychiatric clinical entities and included a control group with “healthy” individuals [17, 25, 26]. As “healthy individuals” were defined those who were not suffering from CU. After meta-analyzing these three studies, among 177,998 CU patients, 56,258 had psychiatric comorbidity (31.61%). Among 996,446 healthy individuals (control group), 45,471 were found to suffer from at least one psychiatric disorder (4.6%) (Table 3).

There were two studies where the number of healthy individuals with psychiatric comorbidity was not determined [27, 28] and another one that did mention the number of CU patients or healthy individuals suffering from specific psychiatric diseases, but not the actual number after adjusting for psychiatric comorbidities [29].

### Main findings

The study by Uguz et al. [26] included a total of 89 CU patients and a control group composed of 64 hospital personnel and their relatives. Forty-four (49.4%) of the patients with CU met the criteria for at least one psychiatric disorder in contrast to the control group where only eight patients (12.5%) suffered from at least one psychiatric disorder.

Atefi et al. [25] aimed to compare the psychological scales in patients with CU with non-dermatological individuals. Thirty patients with CU participated in this study, and 30 individuals without any skin related disorders were enrolled as controls. Nineteen (63.3%) out of the 30 CU patients suffered from psychiatric disorders, whereas this was the case in 14 (46.6%) in the control group.

In 2017 Chu et al. [17] investigated the prevalence, incidence, and comorbidities of CU in the general population of Taiwan. The observed prevalence of psychiatric disorders among 177,879 CU patients was 8.53% (n = 56,195). Among 996,356 healthy individuals (control group), 45,449 (4.56%) were found to suffer from at least one psychiatric disorder.

Staubach et al. [27] in 2006 determined what aspects of life quality are affected and characterize the factors that influence the QoL in CU patients. The study included 100 patients with CU and 96 healthy subjects. Forty-eight CU patients were found to suffer from at least one psychiatric disorder. However, the corresponding number of healthy individuals was not mentioned.

Similarly, the study by Ozkan et al. [28] included 84 CU patients and 75 healthy controls. In this study, 60% of CU patients suffered from at least one psychiatric disorder, but the corresponding number among the healthy control group was not mentioned.

Last but not least, Lapi et al. [29] obtained information on the epidemiology of CU in Italy. The data source was the Health Longitudinal Patient Database. Among 3489 CU patients and 1,714,746 patients without CU, one was found to suffer from excessive alcohol consumption (vs. 2307), eight patients reported insomnia (vs. 5076), 74 patients were suffering from anxiety, dissociative and somatoform disorders (vs. 32,064), and four of them had an acute reaction to stress (vs. 1582), respectively. Since more than one of these symptoms or disorders may coexist in the same patient, it is not clear, and it is not mentioned how many patients suffered from at least one psychiatric disorder so that the relative prevalence could be calculated.

### III. Psychiatric comorbidities in patients with chronic urticaria (studies with control subjects other than healthy individuals)

#### Description of studies

Two studies reported comorbidity of CU with at least one psychiatric clinical disorder and utilized a control group with subjects other than healthy individuals [30, 31].

#### Main findings

Calikusu et al. [31] compared 31 patients diagnosed with psychogenic excoriation (PE) and 31 patients with CU (control group) in terms of comorbid psychiatric disorders. There was a statistically significant difference between the two groups in terms of current major depressive disorder (PE group 58.1%, control group 6.5%, p < 0.01). In the PE group, 45.2% of subjects were diagnosed with obsessive–compulsive disorder (OCD), while the rate of OCD was 3.7% in the CU patients (p < 0.01).

In 2005 Yang et al. [30] studied a total of 75 consecutive outpatients with CU and 133 consecutive patients with tinea pedis who served as the control group. Compared with controls, cases with CU experienced significantly more severe somatic and psychosomatic symptoms.

### IV. Comorbidities with specific psychiatric disorders in patients with chronic urticaria

#### Description of studies

Five studies investigated the CU comorbidity with specific psychiatric disorders [32–36].

#### Main findings

Demet et al. [33] performed a study to determine the prevalence of obsessive–compulsive disorder (OCD) in patients attending the outpatient department of dermatology. Of 166 patients, 41 (24.7%) met the DSM criteria for OCD, among whom 2 had CU (4.9%), while five patients (4%) had CU in the non-OCD group.

Sukan et al. [34] compared 50 vitiligo patients and 50 CU patients with 50 healthy controls to assess sexual dysfunctions. The prevalence of sexual dysfunctions in the vitiligo group were 62.5% (n = 15) in females and 11.5% (n = 3) in males; in the CU group, it was 70.5% (n = 24) in females and 31.2% (n = 5) in males; the rates in the control group were 16% (n = 4) in both females and males. The rates of dysthymic disorders and generalized anxiety disorders were higher in the CU group (46% and 30%, respectively) than the vitiligo group (26% and 6%, respectively).

Chung et al. [32] investigated the relationship between post-traumatic stress disorder (PTSD), stress, psychiatric comorbidity, and personality traits among patients with CU. One hundred patients with CU participated in the study. Sixty patients with allergy (type I hypersensitivity) constituted the control group. Among them, 34% of the CU patients and 18% of the allergic individuals met the diagnostic criteria for PTSD. Patients with CU were 1.89 times more likely to have a current diagnosis of PTSD than allergic individuals.

Bashir et al. [36] aim to determine the frequency of depression among 114 adult individuals that visited dermatology outpatients’ clinics. Two out of 3 CU patients were diagnosed with depression (66.6%).

Finally, in 2016 Tuna et al. [35] included 130 patients with CU and 100 healthy controls in a study that aimed to determine the prevalence and severity of restless leg syndrome (RLS) and to compare the quality of sleep of CU patients with and without RLS. The prevalence (23.8%) of RLS in the CU group was significantly higher than the control group (12%). Similarly, the frequency of poor sleep quality in the CU group was significantly higher than in the control group.

### V. Case reports–case series—psychiatric interventions in patients with CU

#### Description of studies

Very little is known about the effects various psychiatric interventions may have on urticaria, including both psychopharmacological and psychotherapeutic interventions. One case report [37] and two case series [38, 39] were found to mention that treatment of psychiatric disorders with either anti-depressants or anti-anxiety drugs may result in improvement of urticaria.

#### Main findings

In 1995 Hashiro [37] presented a case of a patient with urticaria treated with various combinations of antihistamines (and “antiallergics”) with an only slight improvement of her condition. She presented wheals and erythema despite taking these drugs. Three kinds of psychological tests showed that the patient was highly anxious and depressive, so she was additionally treated with psychotropics and psychotherapy. After a month, the symptoms of both urticaria and psychiatric disorder began to disappear.

The same year Gupta et al. [38] presented two patients with a history of severe CU (corticosteroids were needed regularly to control it) occurring in conjunction with a panic disorder. Both urticaria and panic disorder responded favorably to a course of the selective serotonin reuptake inhibitor antidepressants, fluoxetine and sertraline.

In 2012 Gupta et al. [39] reported five patients with PTSD suffering from CU with or without angioedema. In all patients, CU improved after treatment of the PTSD with trauma-focused psychotherapy intervention.

### VI. Other studies

#### Main findings

Seyhan et al. [40] studied psychiatric morbidity among patients with skin disorders in a dermatology clinic. Of the 636 patients involved in the study, 15.3% were diagnosed with at least one psychiatric disorder. From those with psychiatric morbidity, 25 had CU (25.8%).

In 2014 Perugi et al. [41] explored the prevalence and clinical correlates of general medical conditions (GMC) in a large consecutive sample of patients with Bipolar Disorder. The study sample comprised of 347 patients who met DSM-IV-TR criteria for bipolar disorder type I, bipolar disorder type II, or cyclothymic disorder. Among them, 32 patients (9.2%) were found to suffer from CU.

## Discussion

This systematic review and meta-analysis has investigated the psychiatric comorbidity among patients with CU. The results suggest clearly that the prevalence of psychiatric disorders in patients with CU is significantly higher than in healthy subjects. The overall prevalence of any psychopathology among CU patients after pooling all available data from the meta-analyzed studies was estimated to be 31.6%. This suggests that screening for psychological difficulties/mental health problems among CU patients is a necessity. What is not clear, though, is which might be the benefit of a multidisciplinary approach (allergists/dermatologists and psychiatrists) to control urticaria with or without any additional psychiatric medication or any relevant non-pharmacological psychological or psychiatric intervention. Last but not least, it is not clear if any potential pathophysiological pathways could explain coexistence or sequel from one entity to the other.

### Overall completeness and applicability of evidence

Searching the literature is more than evident that there is limited data about psychiatric comorbidity and factors associated with psychiatric disorders in patients with CU. In 1987 Lyketsos et al. [42] mentioned that patients with CU could develop psychiatric comorbidities. Since then, only a few studies have demonstrated this hypothesis. The development or simply, co-existence of psychopathology and CU seems to be more complicated since the frequency of the differently classified psychiatric disorders does not follow the global prevalence of these disorders in the general population. According to the World Health Organization [43], the global health estimates support depression as the leading mental disorder followed by anxiety. Similar results have been recently demonstrated by Tat TS [44]. However, in this meta-analysis, the sleep–wake disorders were found to be the most common, followed by anxiety and lastly by mood disorders, including depression (Table 4). There could be psychological reasons (i.e., behavioral/social rather than biological factors) why this makes intuitive sense, for instance, sleep disturbances are expected to be more common than in general population due to itch/discomfort, anxiety due to self-consciousness of appearance or uncertainty of flare-ups. It remains to be further examined whether there is a predisposition of CU to these psychiatric entities, or if there are particularities among CU patients that may influence these estimates [CU-specific symptoms and signs, age, ethnicity, gender (most common in females), or if there are just random estimates from the existing studies].

Although psychiatric disorders have been expressed as a potential risk factor of CU [3, 8, 24, 45, 46], the underlying pathomechanisms that may connect these entities have not been clarified yet [11, 45, 47, 48]. Reports suggest that CU may emerge through interactions between the immune and the central nervous system (CNS) [49]. A ‘brain–skin’ connection with local neuro-immuno-endocrine circuitry may underlie the pathogenesis of several allergic and inflammatory skin diseases triggered or aggravated by stress [50, 51]. There seems to be a variety of different pathways, not necessarily mutually exclusive, that could explain how inflammatory dysregulation can affect the brain [52–56]. Peripheral inflammation markers affect the brain without passing the blood-CNS barrier. Proinflammatory cytokines activate the tryptophan-kynurenine pathway, regulate serotonin production together with *N*-methyl-d-aspartate (NMDA) glutamate receptor activity and may also indirectly affect dopamine regulation [57]. The increased inflammation in autoimmune entities may also increase the permeability of the blood-CNS barriers, making the brain vulnerable to cytokines or auto-antibodies. Psychiatric and neurological symptomatology can be induced by brain-reactive antibodies [57–61]. Stress has a vital role in the activation of the immune system and skin by influencing the functions of T cells [57] and can cause abnormal tension on the autonomic nervous system which in turn affects the levels of histamine in plasma and probably in cells [59]. Stress was found to be associated with the activation of the sympathetic, adrenomedullary system, and the HPA axis [57, 62]. In acute stressful situations, both the adrenocortical and medullary systems are activated, leading to an enhanced release of cortisol and catecholamines. However, chronic stress may induce hyporesponsiveness of the HPA axis, whereby cortisol secretion is attenuated and leads to increased secretion of inflammatory cytokines that are typically counter-regulated by cortisol [62]. Thus, chronic stress can result in some inflammatory disorders, such as CU, in which degranulation and mediator release from mast cells and possibly basophils have also been reported [57, 62]. Insomnia itself may further disturb the circadian rhythm of the secretion of cortisol and precipitate the vicious cycle of CU.

A bidirectional relationship between the brain and the immune system also exists [59]. Stress has been shown to suppress or even activate immune system functioning [59, 60]. Stress also has been associated with worse outcomes in immune-related disorders, including infectious diseases and cancer. This fact is of great clinical importance and underlines the vital effect of stress on the immune system and the clinical expression of the disorders [59, 60]. Conversely, several lines of evidence suggest that immune system activation is associated with the development of behavioral symptoms similar to those seen in the context of chronic stress [62]. Nowadays, the activation of the immune system is thought to be one of the main pathophysiological causes of mental health disorders [53, 57, 62]. Abnormal functioning of either HPA axis or the inflammatory process disrupts feedback regulation of both immune and neuroendocrine systems contributing to the development of immune-neuro-psychiatric disorders.

One implication from the pathogenesis model mentioned above is that if the level of stress can be controlled, the impact on the whole chain of biological mechanisms can also be reduced. From this point of view, Gupta et al. [38], as well as Hashiro et al. [37], treated their patients with antiallergic, psychotropic drugs (benzodiazepines and antidepressants) and psychotherapy. The urticaria in all three cases improved. It has not been reported that benzodiazepines and antidepressants have direct effects on the suppression of histamine release. Therefore, it is suggested that neuropeptides from nerve endings may also evoke urticaria. What if patients with CU could benefit from psychiatric treatment? It seems that psychotropic therapy may affect CU control. This evidence needs to be explored.

### Strengths and limitations of this review

The limitations of this work stem primarily from: (i) the lack of intervention studies and randomized control trials; (ii) the heterogeneity of study designs or inappropriate designs (including lack of psychological/psychopathological epidemiological data in the control group, lack of a control group or even comparisons with non-healthy individuals that were used as a control groups); (iii) the heterogeneity among recruited patients and control groups (including age, gender, demographic characteristics, ethnicity, CU severity); (iv) small study sample sizes; (v) different psychometric tools and diagnostic criteria used and, finally, (vi) the lack of studies in other than English language.

### Authors’ conclusion

Current hypotheses, based on the biopsychosocial model, indicate that many organic diseases are multifactorial, contributing to both disease onset and outcome. Whether psychiatric disorders are a potential cause or a consequence of CU remains to be resolved. Further studies should examine the influence of psychiatric disorders and determine the effects of appropriate psychiatric interventions, including pharmacotherapy or psychotherapy on the course of CU. Till then, our findings call for mental health evaluations of patients with CU in routine clinical practice. Patients with CU should be referred, investigated and treated for any mental health disease, a cooperation between allergists/dermatologist and psychiatrists that, teleologically, is expected to improve both health and QoL in these patients.

## Data Availability

All data generated or analyzed during this study are included in this published article.

## Abbreviations

CU: chronic urticaria or chronic idiopathic urticaria or chronic spontaneous urticaria
DSM: Diagnostic and Statistical Manual
PNS: peripheral nerve system
ICD: international classification of diseases
OCD: obsessive–compulsive disorder
RLS: restless leg syndrome
PTSD: post-traumatic stress disorder
CNS: central nervous system
HPA axis: hypothalamic-pituitary-adrenocortical axis
